# Nanotechnology-based advances in the efficient delivery of melatonin

**DOI:** 10.1186/s12935-022-02472-7

**Published:** 2022-01-29

**Authors:** Mohammad Mirza-Aghazadeh-Attari, Ainaz Mihanfar, Bahman Yousefi, Maryam Majidinia

**Affiliations:** 1 Student Research Committee, Tabriz University of Medical Sciences, Tabriz, Iran; 2 Department of Biochemistry, Faculty of Medicine, Urmia University of Medical Sciences, Urmia, Iran; 3 Immunology Research Center, Tabriz University of Medical Sciences, Tabriz, Iran; 4 Solid Tumor Research Center, Cellular and Molecular Medicine Institute, Urmia University of Medical Sciences, Orjhans Street, Resalat Blvd, Urmia, Iran

**Keywords:** Melatonin, Nano-delivery, Chitosan, Liposomes, PLGA, Solid lipid nanoparticles

## Abstract

*N*-[2-(5-methoxy-1*H*-indol-3-yl) ethyl] or simply melatonin is a biogenic amine produced by pineal gland and recently recognized various other organs. Because of a broad range of biological function melatonin is considered as a therapeutic agent with high efficacy in the treatment of multiple disorders, such as cancer, degenerative disorders and immune disease. However, since melatonin can affect receptors on the cellular membrane, in the nucleus and can act as an anti-oxidant molecule, some unwanted effects may be observed after administration. Therefore, the entrapment of melatonin in biocompatible, biodegradable and safe nano-delivery systems can prevent its degradation in circulation; decrease its toxicity with increased half-life, enhanced pharmacokinetic profile leading to improved patient compliance. Because of this, nanoparticles have been used to deliver melatonin in multiple studies, and the present article aims to cumulatively illustrate their findings.

## Introduction

Recent advances in medicine have led to the introduction of numerous agents to treat multiple human pathologies, such as various types of cancers, degenerative disorders, and infectious diseases. This advance has led to hopes of targeting important cellular pathways, which are involved in the process of aging and cellular regeneration [1]. Therapeutic agents need to be delivered to specified locations in order to maximize their effects on the target organs, and to limit potential side effects to peripheral tissues [2–4]. In addition, this means of delivery should be easy to produce on industrial scales and cost-efficient to administer, should be safe and biodegradable, and more importantly, should not have unwanted interactions with the agent it is supposed to deliver [5].

Melatonin is a molecule with a short half-life and swift elimination from circulation, and because of its molecular properties, has a limited absorption from mucosal and dermal surfaces [6]. More so, because melatonin can affect receptors on the cellular membrane, in the nucleus and can act as an anti-oxidant molecule, some unwanted effects may be observed after administration. As mentioned, the emergence of new biomaterials and drug delivery systems has enabled scholars to experiment with melatonin in order to find solutions to its limitations for routine clinical application in fields other than sleep medicine [7]. One group of new delivery methods, and probably the most widely studied, is the use of nanoparticles to deliver various agents [8]. Nanoparticles usually size under 100 nano-meters and are composed of various biodegradable products, polymers, lipids, metals and other compounds [9]. Further, nano-particles have all or most of the aforementioned characteristics necessary for novel delivery techniques [10]. Because of this, nanoparticles have been used to deliver melatonin in multiple studies, and the present article aims to cumulatively illustrate their findings.

### Novel technologies in drug delivery

Designing therapeutics is a process with many challenges. Even if the designing a drug that modulates the action of a particular biological target in vitro is overcome, selective delivery to that target in vivo presents a major barrier; and the need to deliver a pharmacological dose of a drug to a targeted site with high efficacy, using a route that will facilitate patient compliance, is of critical importance for effective and safe disease management [11]. However, the established use of new methods (macromolecules, nanostructures, and etc.) to encapsulate or conjugate drugs can provide improved delivery, and stands to enable better therapeutic outcomes [12, 13]. Drug delivery systems (DDSs) improve the administration and efficacy of pharmaceutical compounds including antibodies, peptides, vaccines, drugs and enzymes [11, 14]. In one category, the recently discovered new drug delivery systems include lipid, protein and polymer technologies with better lipid distribution in the body, prevention of drug degradation from external environment and reduced rate of drug clearance [15]. In another type of classification, seven categories of DDS including microsphere-depots, tumor-targeting nanoparticles, transdermal patches, advanced oral pills, inhalers, implants and antibody–drug conjugates are highlighted [14]. One of the most important applications of these technologies is in the cancer treatment methods. Despite the development of numerous chemotherapeutic drugs with desirable anticancer effects in preclinical trials, their effectiveness in clinical settings remains disappointing [16–19]. This is partially attributed to the delivery and transport limitations of anticancer drugs in tumor interstitium. Especially in solid tumors there are several barriers posed by the tumor microenvironment [20]; but some of these problems are solved using the DDSs. The new DDSs are discovered with the aim to resolve solubility problems, prevent external environment issues on the drug such as photo degradation and pH changes [21], make the drug more lipid soluble so that it can easily cross lipid barriers in the body and achieve desired concentration at the desired location for maximum therapeutic effect [22, 23]. The therapeutic benefits of the new DDSs consist of increased efficacy of the drug, site specific delivery, reduced side effects, increased patient compliance and convenience and reduced healthcare costs [24, 25]. Some of the novel DDSs consist of transdermal drug delivery systems, colloidal drug carrier systems (liposomal delivery systems, nanoparticulate delivery systems, micelles and dendrimers), variable release delivery systems, implantable delivery systems and nasal delivery systems [26]. We have explained some of these systems and other new systems below.

### Melatonin: a hormone with wide effects

*N*-[2-(5-methoxy-1*H*-indol-3-yl) ethyl] or simply melatonin is a biogenic amine which is produced in both plants and animals and in mammals it is produced by the pineal gland. Melatonin is produced in a stepwise process, with l-tryptophan undergoing hydroxylation, turning it into hydroxyl-tryptophan, which then undergoes decarboxylation, creating serotonin which eventually is processed by serotonin *N*-acetyl transferase and hydroxyindole *O*-methyl transferase, and transforms to melatonin [27, 28]. It will be mentioned in the next paragraphs that melatonin is used as medication in some conditions and is being considered for many more. Because of this the pharmacokinetics of this agent is of significant importance. A comprehensive systemic review by Groth Harpsøe et al. suggested the following characteristics for melatonin, a dosage ranging between 0.3 and 100 mg as an oral agent or intravenous injection, C max ranging from 72.1 to 101.163 pg/ml, T max ranging from 28 to 126 min and a bioavailability of 9 to 33% [29]. Although melatonin has many uses, because of the less favorable pharmacokinetic characteristics of it, its use is currently limited. One method of increasing the future relevance of melatonin in current medicine, is establishing new delivery methods [30].

### Nanotechnology for melatonin delivery

#### Selenium based NP

**Fig. 1:**
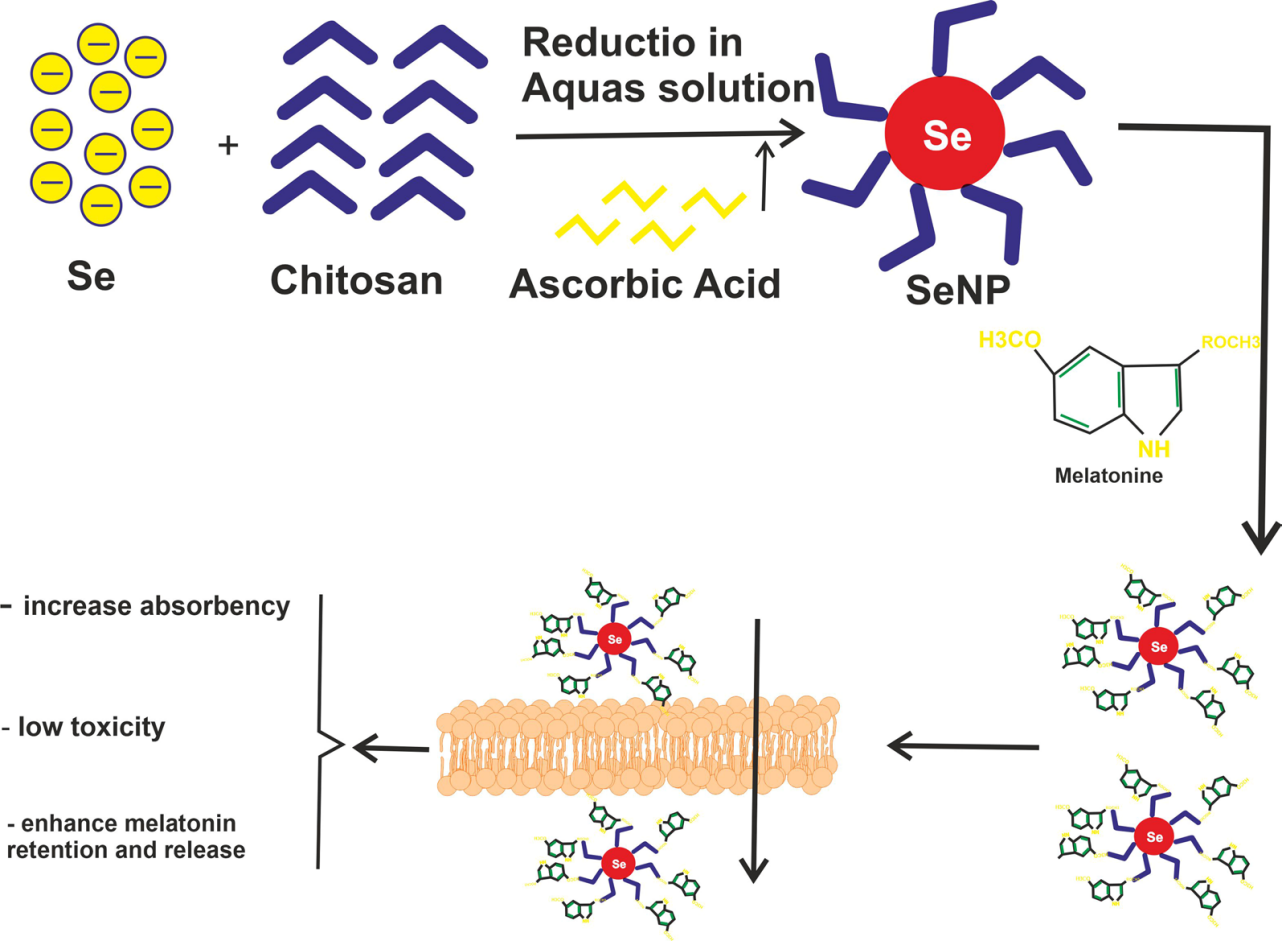
The schematic illustrates provided show melatonin selenium-based nanoparticle and its effects

Selenium is an essential trace element, which is used to build Selenoproteins, proteins with prominent enzymatic activity [31]. Selenium is found in both organic and inorganic forms, but these forms have limited absorbance from the gastro-intestinal tract, and most importantly could have possible toxic effects in high doses. These limitations have been the rationing behind the development of selenium nano-particles. Selenium nano-particles (SeNPs) are synthesized by multiple methods, including the chemical reduction method, the sovothermal method, microwave assisted method and the use of specific microscopic organisms to synthesize these NPs. The most notable method used is the chemical reduction method where selenium is initially mixed with an aqueous solution of a polysaccharide, namely chitosan, and then ascorbic acid is added to complete the formation of SeNPs [32, 33]. SeNPs have been shown to have profound anti-cancer effects, both as a monotherapy and combined therapy as a drug delivery instrument in cancer cell lines. For example, a combination of doxorubicin-selenium nanoparticle-liposomes was shown to increase apoptosis in tumor cells compared to each of the therapies administered alone [34], further, administration of selenium itself could increase ROS production, induce DNA damage and disrupt the DDR sequence, consequentially leading to apoptosis [35]. The beneficence of combining melatonin and SeNPs has been shown in multiple studies. Wang et al. showed that a combination of these two agents significantly decreased damaged caused by immunologic reactions following the administration of bacillus Calmette-Guerin to liver cells in mice. This was achieved by inhibiting the initial response after BCG administration, which was composed of an increase in the levels of nitric oxide (NO), NF-alpha and IL-1beta [36]. The same team of scholars also showed that this combined treatment efficiently decreased the damaging effects of ROS after exposure to BCG, by increasing the activity of glutathione peroxidase, a seleno-protein and decreasing lipid peroxidation (Fig. 1) [37].

### Chitosan based NPs

Chitosan is a natural compound found abundantly in crustacean shells of multiple species, most notability shrimps. This agent is made from deacetylated chitin, and chitosan based NPs are prepared synthetically using methods such as micellization, ionic gelation and Emulsification [38]. Chitosan based nanoparticles have multiple usages in tissue engineering, gene delivery and cancer imaging [38, 39]. Studies have focused on the capacity of chitosan as a route of transdermal or trans-mucosal delivery. In a study by Hafner et al. it was shown that Lecithin/chitosan nanoparticles were indicated in transdermal use in porcine skin. They showed that these nanoparticles could be applied in concentrations up to 200 µg/mL without causing a significant permanent damage to human keratinocytes and fibroblasts, two important cells of the human skin [40]. More so, in another study Hafner et al. showed that these NPs could be made using trehalose as a lyophilisation agent, which conferred it a suitable glass transition temperature and a long period of preservation time (up to 7 months) [41].

In a study by Shokrzadeh et al. chitosan was used to deliver melatonin to HepG2 cancer cells undergoing treatment with etoposide, a topoisomerase II inhibitor. Etoposide is a genotoxic agent. Melatonin was administered to assess its capability in reducing DNA damage and ROS formation resulting from Etoposide administration. Comparing the control group undergoing etoposide treatment alone and the intervention group undergoing melatonin delivered by chitosan showed that in the latter group the cells experienced alleviated DNA damage and oxidative stress, and an increased capacity to utilize glutathione as an anti-oxidant. The most significant results were obtained in a group of cells which underwent melatonin treatment 24 h prior to etoposide treatment [42]. Topal et al. utilized a melatonin/2-hydroxypropyl-β-cyclodextrin (HPβCD) inclusion complex loaded onto scaffolds of chitosan, to inhibit growth and proliferation of MG-63 osteosarcoma cells. In this study, the scaffold was prepared by the freeze-drying method. The results showed that melatonin, in a concentration of 9 m-M, which was achieved by the utilization of the scaffold, was able to significantly increase cell death, and reduce the proportion of the cells in the G2/M phase [43]. Yadav et al. investigated the effect of chitosan/tripolyphosphate (TPP) loaded melatonin NPs on the malignant U87MG cell lines, both cultured alone and co-cultured with the non-malignant human HEK293T cells. They used the ionic gelation method to create TPP NPs and after administration to the cultures, found that these NPs had increased uptake compared to free melatonin, and in the co-culture, the uptake was more significant in the malignant cell line. Cells were incubated for 24. 48 and 72 h to assess their reaction to treatment, and free melatonin was used as a control factor. It was found that nano-delivered melatonin was able to induce cell death in 78% of cells after 24 h, while free melatonin was able to induce cell death in only 58% of the cells. More so, cell viability reached a minimum in free melatonin administration, but continued to decline in the nano-delivery group. These toxic effects on malignant cells were accompanied by minimum damage to non-malignant cells, both in co-culture and in an isolated culture [44]. As mentioned before, Hafner et al. conducted multiple studies regarding lecithin/chitosan nanoparticles and their suitability as trans-mucosal delivery agents. These scholars also conducted a study where melatonin was coupled with lecithin/chitosan nanoparticles and administered to Caco-2 colorectal cancer cells. The results showed that this method was efficient in delivery of melatonin across mucosal surfaces, as the delivery agent prepared enough entrapment capacity for melatonin and because of the electrical charge of it, enabled sufficient muco-adhesion. They also concluded that the best formulation for a lecithin/chitosan was achieved when S45 lecithin was used and the ratio of lecithin to chitosan was 20–1 [45]. El-Gibaly et al. demonstrated that chitosan microcapsules were an efficient vehicle to transmit buoyant (B) melatonin to rat liver cells being treated with aflatoxin B1, a toxic agent causing severe damage and apoptosis. They found that utilizing the aforementioned method, a 36.90–56.23% encapsulation percentage was received coupled with a t_1/2_ up to 5 h. In the in vitro examination, administration of melatonin using the aforementioned particles resulted in the shifting of the balance between anti-oxidant molecules and oxidative products which caused a significant decline in apoptosis rates, which were shown was a decline in active caspase 3 levels and histopathologic findings [46]. Blazevic et al. examined the effects of melatonin loaded on to chitosan- lecithin nano-particles, on an in-vitro wound model. In an in-vitro study performed on HaCaT Human keratinocyte cell line. They found that melatonin NPs increased re-epithelization of wound models, with significant differences in just 24 h after administration [47].

**Fig. 2:**
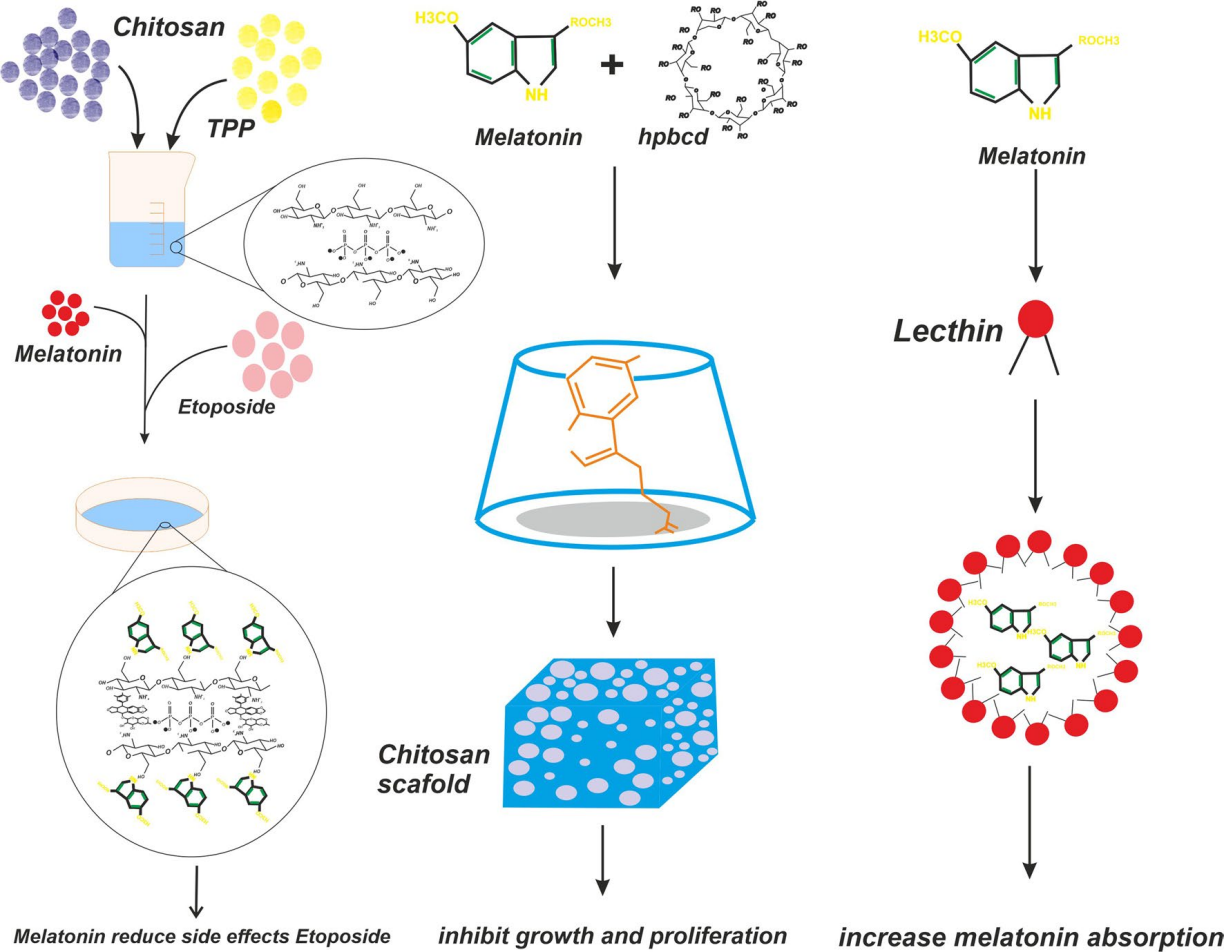
The schematic diagram provided reveals melatonin chitosan nanostructures and its functions

One interesting function of melatonin is its ability to decrease intra-ocular pressure in various animals and disease models, and various scholars have experimented with different delivery methods in this regard. In a study by Hafner et al., lecithin/chitosan and Pluronic^®^ F127/chitosan NPs were used to deliver melatonin to an in vitro model of HCE-T epithelial cells. The later, was significantly smaller compared to lecithin/chitosan NPs, but showed increase permeation, compared to lecithin/chitosan NPs ability to adhere to mocusal surfaces and achieve sustained drug delivery. None of these NPs had any toxic effects on the cells, and both of them showed suitable characteristics as pre-corneal delivery agents (Fig. 2) [48].

### Solid lipid NPs and nanostructured lipid carriers

Introduced in the last decade, solid lipid NPs (SLNs) are lipid emulsions, where a solid lipid is present [49]. These agents have unique abilities in penetrating distant cites of an organism, contained by hydrophobic barriers, such as the CNS. Further, these agents have an optimal binding to lipophilic medications and also to hydrophilic ones. More so, studies have shown that SLNs have beneficial capabilities in achieving controlled release rate with different medications [50]. Priano et al. investigated the pharmacokinetics of melatonin administered via SNLs. They found that oral and transdermal administration of this combination significantly increased the serum levels of melatonin on a daily basis, as 3 mg melatonin administration with SNLs resulted in plasma levels of melatonin which were kept above 50 pg/ml for about 24 h. Transdermal administration of melatonin incorporated to SNLs generated a half-life of absorption equal to 5.3 ± 1.3 and a half-life of elimination of 24.6 ± 12.0 h, which enabled a sustained release for melatonin, mimicking its natural secretion in the body [51]. In one study, Rezzani et al. investigated the effects of melatonin on cyclosporine A induced toxicity in cardiac tissue of Wistar rats. They administered melatonin in two regimens, one consisting of 1 mg/kg/day intra-peritoneal melatonin and 15 mg/kg/day sub-cutaneous cyclosporine A for 21 days, and the second regimen consisting of the same amount of melatonin incorporated on to SNLs. They witnessed that cyclosporine A had pro-apoptotic effects on cardiac cells, and melatonin was able to antagonize these adverse effects. Administration of melatonin alone did not result in a marked anti-apoptotic response, but introduction of SNLs loaded with melatonin significantly inhibited apoptosis. The authors suggested that when melatonin was bound to SNLs, cells utilized endocytosis to uptake the large SNL particles, thus up taking melatonin. Then melatonin acted as an anti-oxidant molecule and exerted its effect by acting as a scavenger molecule, rather than activating its specific receptors (MT1 and MT2) and the downstream signaling pathways [49]. It is noteworthy to mention that melatonin may indeed have some protective role utilizing these receptors, but the effect seems to be inferior to that seen when melatonin is active as an intracellular scavenger molecule [52]. Sabzichi experimented with nanostructured lipid carriers (NLCs) and used them to deliver melatonin to MCF-7 breast cancer cells undergoing treatment with tamoxifen. IC50 values for melatonin and tamoxifen were 1.3 ± 0.4 mM and 30.7 ± 5.2 μM, respectively. Co treatment resulted in a double fold increase in rates of apoptosis this was also coupled with reduced expression of survivin, an anti-apoptotic molecule [53]. Marepally et al. used several kinds of novel lipids in order to facilitate the transdermal delivery of multiple agents, including melatonin. They found that 1-Di-((Z)-octadec-9-en-1-yl) pyrrolidin-1-ium iodide (Cy5) significantly increased the transdermal delivery of melatonin, and cy5 lipid ethanol drug nanoparticles even made the permeability better, and had sustain release characteristics [54].

### Liposomes

**Fig. 3:**
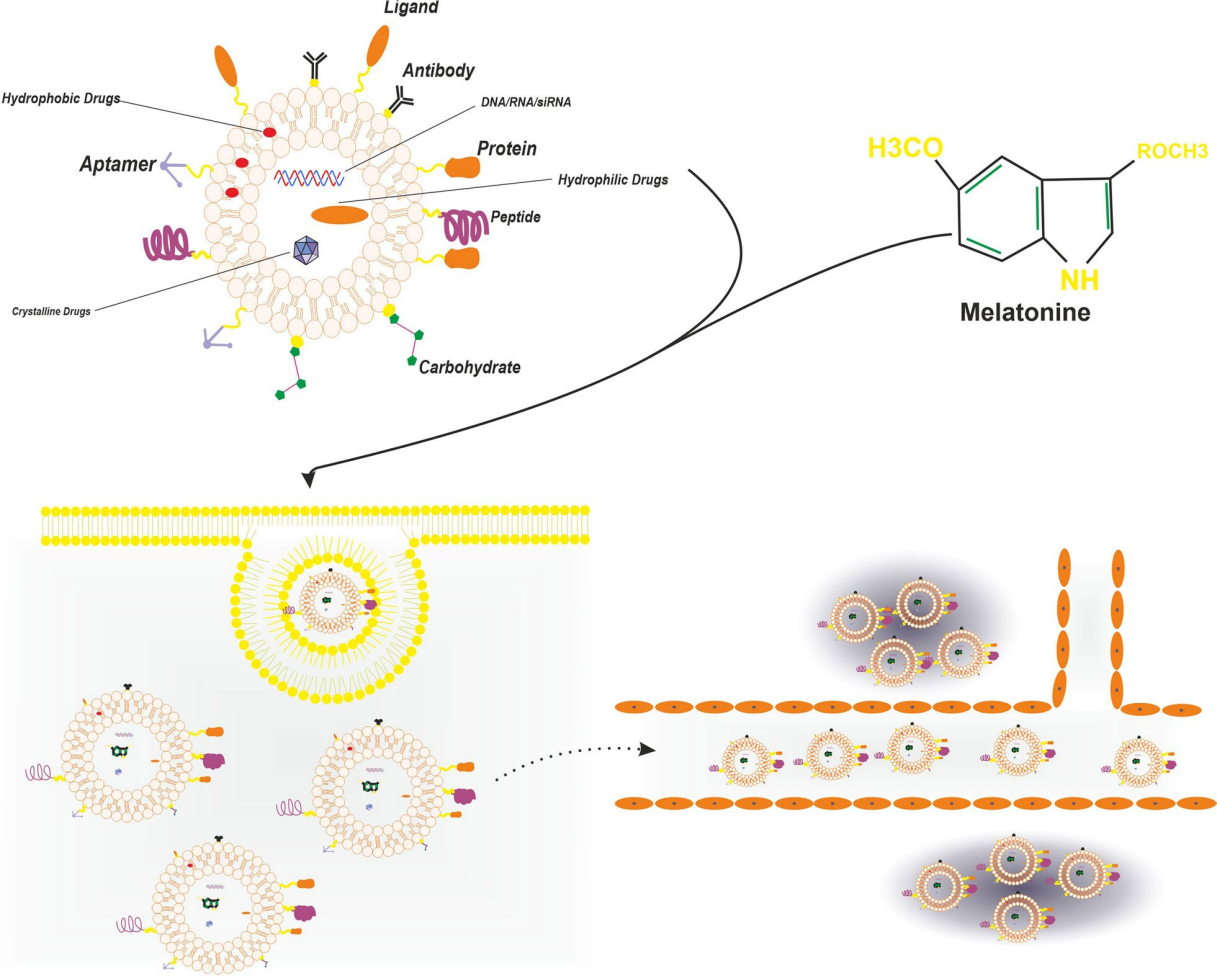
The figure shows the liposome as delivery vehicle for melatonin, liposome increase the transdermal delivery of melatonin and also increase distribution of melatonin in multiple part of body like cornea as fragile site

Liposomes are composed of two layers of natural or man-made phospholipids, which can be made spontaneously in aqueous environments. Drug loading into liposomes can be done by methods such as formation of liposomes in an already saturated environment, pH gradient methods, specially used for hydrophobic agents with a primary or secondary amine, the use of solvents and solvent exchange, loading ultra-high concentration of drugs to reach precipitation, utilization of supercritical solution technology and use of encapsulating polyanions [55–57]. Liposomes are used to deliver numerous agents, ranging from micro-RNAs to anti-cancer drugs and conventional chemotherapy agents [55, 58]. Liposomes have also been used in multiple studies to enhance the delivery of melatonin. Much interest has been given to the use of liposomes as transdermal delivery vehicles of melatonin. Dubey et al. described ethanolic liposomes as a method to increase the transdermal flux of melatonin, decrease lag times, increase dermal lipid mobility and finally increased permeation of melatonin [59]. Dubey et al. also showed that elastic liposomes, a subgroup of liposomes made from hosphatidylcholine and edge activators to grant liposomes further membrane flexibility, were more efficient than normal liposomes, regarding Entrapment percentages, release rates, transdermal flux, Permeability and Diffusion coefficients and lag time [60]. In a study by Sana et al. nanoencapsulated and liposomal forms of melatonin were introduced to rats under exposure to sodium fluoride, a widely used chemical toxin. This toxin generates profound amounts of ROSs, which target the lungs and the liver, inducing the expression of TNF-α, TGF-β and reducing the amount of anti-oxidant enzymes in the cells. Melatonin administered via the two aforementioned methods was able to significantly reverse the effects of sodium fluoride two hours after its exposure, and increase the anti-oxidative capacity of cells undergoing treatment [61]. One important function of liposomes has been the ability of drug delivery to fragile cites, without causing irritation of damage to the organ. Delivery to the cornea is one example. One particular condition is Glaucoma, which is a condition characterized by high ocular pressure, and is in need of continuous administration of topical agents which decrease the production of the aqueous humour. These topical agents are not without their own harms, as they may contain toxic preservatives or allergens [62]. In a study by Quinteros et al. a combination of liposomes containing the hypotensive melatonin analog 5-methoxycarbonylamino-N-acetyltryptamine and mucoadhesive substances such as sodium hyaluronate or carboxymethylcellulose were used to treat high ocular pressure in rabbit eyes. The results showed that the aforementioned combination decreased the intra ocular pressure by 39.1 ± 2.2%, which was significantly higher than liposomes with other agents. Further, this reduction in intra-ocular pressure continued for 8 h [63]. One important pathologic process in multiple human pathologies is the formation of lipid peroxidation products, which act as stable sources of oxidative stress in tissues [64]. Schaffazick et al. have shown that melatonin nano-vesicles, could have protective roles against lipid peroxidation and the secondary damage inflicted by them (Fig. 3).

### PLGA, PLA nanoparticles and PEG

Altindal et al. demonstrated the possibility of using poly (d,l-lactide-*co*-glycolide) (PLGA) nanoparticles as a method of delivery. PLGA nanoparticles are biodegradable and biocompatible polymers which have been FDA approved for parenteral delivery and show sustain release qualities [65]. It this study, melatonin loaded PLGA micro and nanoparticles were made using the emulsion-diffusion-evaporation method. Drug release assays showed that after 40 days of administration of these particles to an in vitro culture of MG63 osteosarcoma cells, 70% of the melatonin content was released. These nanoparticles had an uptake percentage of 60% in the first day, with a rapid uptake happening in the first 5 h. Analysis of the culture showed that melatonin administration equaling to 1.7 micro-Grams showed obvious inhibitory effects on cancer cells, coming short of inducing apoptosis [65]. PLGA nanoparticles were also used to utilize the anti-oxidant effects of melatonin, in a study which was conducted by Martins et al. they showed that these nanoparticles reduced oxidative damage in erythrocytes, ultimately preventing hemolysis [66]. As noted, before, one novel function of melatonin has been its use in decreasing intra-ocular pressure, thus acting as an anti-glaucoma agent. In a study by Musumeci et al. PLGA and poly (ethylenglycole) (PEG) nanoparticles were loaded with melatonin and administered to rabbits. This intervention was then compared to the administration of melatonin as an aqueous solution. It was found that the NPs generated the most significant decline in IOP in a time period of 8 h, and the maximum reduction was 5 mmHg, which could be regarded as clinically significant [67]. Ma et al. studied the effect of melatonin loaded PLGA-mPEG NPs on adipose-derived stem cells which were transplanted in an infarcted heart model. The rationale behind exposing stem cells to melatonin in ischemic conditions is that ischemia itself and the reperfusion happening after it, both are massive sources of ROS, and that melatonin, as an anti-oxidant molecule could benefit cells in establishing themselves in the new environment. In this study, an in vitro investigation revealed that administration of NPs containing melatonin reduced the formation of p53-cyclophilin D complex and stabilized the mitochondria, thus rescuing stem cells from injury. In vivo investigations showed that nano-administration of melatonin, was superior to melatonin injection in rats undergoing cardiac ischemia which were implanted with stem cells [68].

The ability of melatonin as an anti-oxidant has led researchers to consider it as a possible treatment for sepsis, a condition with an elevated amount of ROS which causes cell damage in important organs such as the liver, and with this causes morbidity and mortality [69]. Chen et al. investigated the role of PEG and poly (propylene sulfide) (PPS) as drug delivery systems in an in vivo model of sepsis, to evaluate their effect on hepatic cells. Treatment with nano-delivered melatonin resulted in a more significant decline in inflammatory cytokine levels and lipid peroxidation in hepatic cells compared to free melatonin. Furthermore, nano-delivered melatonin reduced the levels of hepatic enzymes (ALT and AST) in mice, showing a reduced damage to the hepatic tissue. Investigation of NF-KB signaling, revealed that phosphorylation of p65 of NF-KB had markedly decreased in the group receiving nano-treatment, compared to the group which only received free melatonin [70, 71].

### Other strategies

Nano-capsules are small shell-like structures which are made from polymers. Generally, nano-capsules are composed of a core which can be liquid based or polymer based, and a polymeric membrane [72]. Nano-capsules have a lower polymer use compared to other nano-delivery methods, and have a superior drug stability because of the existence of a core protected by the outer layers. Further, nano-capsules are relatively resistant to environmental factors such as dramatic changes in the PH levels [73, 74]. Komninou et al. examined the effects of two types of melatonin loaded nano-capsules, one with a lipid core and another with a polymeric core, on a Bovine Embryo Culture Model. They examined the rates of apoptosis and genes involved in the process, ROS production and embryonic development. It was shown that melatonin-loaded lipid-core nano-capsules in concentrations of 10 M, in compare to polymer core nano-capsules, were able to increase hatching rates, reduce apoptosis, downregulate the expression of genes such as BAX, CASP3, and SHC1 and upregulating the expression of CAT and SOD2, ultimately leading to increased survival and reduced ROS formation [75]. Hoffmeister et al. experimented with a hydrogel formation containing spray dried-melatonin loaded nano-capsules. They found that use of the aforementioned technique; significantly delayed melatonin release from NPs compared to water-based solutions containing the same NPs and delayed permeation in a model of pig skin. Further, the hydrogels showed an increased release in more distant intervals of administration, having an optimal sustain release capacity [76]. In another study, Schaffazick et al. experimented with polymeric nano-capsules coated with polysorbate 80, and investigated their efficacy in delivery of melatonin, as an anti-oxidative agent to the brain and liver, to inhibit lipid peroxidation. In an in vivo study conducted on mice, they showed that injection of a melatonin aqueous solution to different sites in the peritoneum did not cause any significant anti-oxidative effect and was not able to inhibit lipid peroxidation. In contrast, administration of melatonin loaded in nano-capsules significantly inhibited lipid peroxidation in multiple anatomical sites and increased the total anti-oxidant reactivity in the hippocampus. The dose of melatonin used in this experiment was 10 mg/kg, and in this dosage nanoparticles were clearly superior to an aqueous solution [77].

Starch microspheres are another delivery technique with promising outlooks for transdermal and trans-mucosal delivery. Micro-spheres resemble nan-capsules, but they contain more residual structures such as polymers, but share the same suitable drug delivery characteristics including the insurance of sustained release of agents and easy administration. But they lack the stability that micro-capsules offer [78]. Lee et al. made melatonin loaded micro-spheres using dual walled chitosan and sodium alginate beads and utilizing the emulsion melting/cooling method. The scholars declared that using the latter method was inexpensive, and resulted in spheres with optimal drug delivery abilities [79]. Mao et al. were able to create Melatonin starch microspheres sizing between 30 and 60 micro-ms, using the emulsification-crosslinking technique. They used these spheres in the nasal cavity and found that this method increased the t_1/2_ time from 5.6 min for pure melatonin to 12.3, and also increased the longevity of the agent present in the nasal cavity, from 30 to 80% [80].

One other intriguing new method of drug delivery is using hydroxypropyl methylcellulose (HPMC) made meshes or tablets. These products produced from HPMC are easy to manufacture, are safe and have suitable drug delivery characteristics [81]. Lee et al. produced HPMC tablets, using HPMC, melatonin and agents such as Avicel, Primojel, Polyplasdone and Ac-Di-Sol as disintegrates and Mg-stearate, Cab-O-Sil and Talc as Lubricant/Glidant. It was found that higher viscosities and particle sizes of HPMC (100 cps compared to 300 and 4000) resulted in decreased drug release rate. Use of different additives and lubricants, did not result in any significant change in drug release properties of the gel. But adding Avicel and Mg-stearate to high concentration HPMC did result in a decline of zero drug release period (from 10 to 4 h). Further, use of coating methods was able to increase this interval to more than 10 h. In conclusion, the authors suggested that manipulating the various components of HPMC tablets could bring about the suitable mixture for oral delivery of melatonin using HPMC products [82].

Iron oxide Magnetic Nano-composite Particles (MNPs) are novel biomaterials invented to deliver drugs into cells and also have the potential to manipulate cells by their magnetic characteristics, also enabling them to act as a contrast material for imaging techniques such as magnetic resonance imaging [83]. In a study by Xie et al. single emulsion solvent extraction/evaporation method was used to make Melatonin-MNPs, which were administered to MCF-7 breast cancer cells in an in vitro study. The NPs had an optimal up-take by the cells, and adding a magnetic field to the cell medium, caused an inductive heating reaction, both acting as an independent cyto-toxic agent and also as a method to increase sustainable release in melatonin carrying particles [84].

Massella et al. used two other novel delivery systems, the Cotton Fabrics with Polycaprolactone Nanoparticles to deliver melatonin as a transdermal medication. These particles then applied to a dermal patch, which resulted in an encapsulation percentage of 80%. More so, distribution analysis for Cotton Fabrics showed a continuous and controlled release rates [85].

## Melatonin loaded NPs in clinics

As mentioned before, melatonin is involved in multiple processes that are key factors in numerous human pathologies. Because of this melatonin is readily used in conditions such as insomnia, jet lags and work shift sleep disorders, and is being evaluated in clinical trials for a wide array of conditions including multiple sclerosis, hypertension, sepsis, neoplastic conditions [86, 87]. Currently, the most commonly used forms of melatonin are tablets, capsules and occasionally topical substances [88], and the concept of using nanoparticles for delivery of melatonin is still in its early steps, with studies focusing on in vitro delivery on various models of human conditions [89]. Of all of nano-delivery methods, only limited numbers are investigated regarding the delivery of melatonin and complete classes of vehicles such as dendrimers are not investigated. Further, safety issues and cost analysis for human administration are also largely missing.

## Conclusion

In this review the importance of novel drug delivery methods was noted and it was discussed that nano-particles were indeed one of the most promising agents in this regard. It was also discussed that nanoparticles had important characteristics making them ideal for safe and efficient delivery of both hydrophobic and hydrophilic agents, such as melatonin. This article cited the multiple functions of melatonin in human pathologies and its effect on cell signaling pathways which define its importance in many cellular processes. Then the application of multiple nano-delivery systems was shown in melatonin delivery and it was discussed that these novel delivery systems enable suitable drug delivery and other needed characteristics. It was also shown that the application of drug delivery methods ranged from neoplastic conditions, to degenerative processes, regenerative medicine, oxidative conditions and more. At last it was noted that use of melatonin, as an agent with numerous merits, and novel drug systems was limited to in vitro and animal studies, and more evidence is needed to conclude the efficacy in humans. Regardless of this lack of evidence n human subjects, if not all, most studies currently performed signal the importance of drug delivery methods in the future of medicine, and delivery of melatonin.

## Data Availability

Not applicable.
